# A Preliminary Report; Etiology, Pathology and Treatment of Pellagra; a New Theory, a Specific Cause

**Published:** 1911-06

**Authors:** George C. Mizell

**Affiliations:** Atlanta, Ga.; Formerly Associate Professor of Physiology and Gastro-Enterology of Atlanta College of P. & S.; Formerly Gastro-Enterologist to Wesley Hospital


### Journal-Record of Medicine

Successor to Atlanta Medical and Surgical Journal, Established 1855, and Southern Medical Record, Established 1870.

**OWNED BY THE ATLANTA MEDICAL JOURNAL CO.**

**Published Monthly**

*Official Organ Fulton County Medical Society, State Examining Board, Presbyterian Hospital, Atlanta, Birmingham and Atlantic Railroad Surgeons' Association, Chattahoochee Valley Medical and Surgical Association, Etc.*

EDGAR G. BALLENGER, M. D., Editor.
BERNARD WOLFF, M. D., Supervising Editor.
A. W. STIRLING, M. D., C. M., D. P. H., J. S. HURT, B. Ph., M. D.
GEO. M. NILES, M. D., W. J. LOVE, M. D., (Ala.); Associate Editors.
E. W. ALLEN, Business Manager.

COLLABORATORS

Dr. W. F. WESTMORLAND, General Surgery.
F. W. McRAE, M. D., Abdominal Surgery.
H. F. HARRIS, M. D., Pathology and Bacteriology.
E. B. BLOCK, M. D., Diseases of the Nervous System.
MICHAEL HOKE, M. D., Orthopedic Surgery.
CYRUS W. STRICKLER, M. D., Legal Medicine and Medical Legislation.
E. C. DAVIS, A. B., M. D., Obstetrics.
E. G. JONES, A. B., M. D., Gynecology.
R. T. DORSEY, Jr., B. S. M. D., Medicine.
L. M. GAINES, A. B., M. D., Internal Medicine.
J. N. LeCONTE, M. D., Disease of the Stomach and Intestines.
L. B. CLARKE, M. D., Pediatrics.
EDGAR PAULIN, M. D., Opsonic Medicine.
THEODORE TOEPEL, M. D., Mechano Therapy.
R. R. DALY, M. D., Medical Society.
A. W. STIRLING, M. D., etc., Diseases of the Eye, Ear, Nose and Throat.
BERNARD WOLFF, M. D., Diseases of the Skin.
E. G. BALLENGER, M. D., Diseases of the Genito-Urinary Organs.

VOL. LVIII. JUNE, 1911 No. .3

### A PRELIMINARY REPORT; ETIOLOGY, PATHOLOGY AND TREATMENT OF PELLAGRA; A NEW THEORY, A SPECIFIC CAUSE.

BY GEORGE C. MIZELL, M.D. PH.D., ATLANTA, GA.

*Formerly Associate Professor of Physiology and Gastro-Enterology of Atlanta College of P. & S.; Formerly Gastro-Enterologist to Wesley Hospital.*

In presenting this report to the medical world I do so with a full knowledge of its far-reaching import. As a caution I have before me the criticism of Prof. Lombroso who justly, no doubt, calls attention to the harm resulting from many visionary and obscure theories that have been advanced regarding the etiology of pellagra.

In search of a cause for pellagra men have spent years and great sums of money. Based upon the maize theory governments have established institutions for treatment. Laws have been enacted regulating the sale of spoiled corn. These preventive and paliative measures have been in operation for years with little effect. In the last decade our own country has been attacked and the rapidity of the advance now amounts to a scourge. An official of our Board of Health states that there are fifty thousand cases in the State of Georgia, at this date. We all ask the cause and relief. No one has heretofore answered. I deeply regret that the answer involves an attack upon an article of diet, the use of which is of vast importance agriculturally and financially to our Southern States, and to a degree the whole world.

Cotton seed oil now constitutes a great per cent. of our fatty foods. Oil of Sesame occupies a similar position in the East. These oils are the source of much wealth in large sections.

We know that olive oil has been eaten from time immemorial—that it is adulterated with cheaper oils cannot be doubted. (The character of the oil used as an adulterant determines the wholesomeness of the adulterated oil.)

In order to open a way for another theory explaining the presence of pellagra in the United States and its presence in Italy and France for years, I will try to disprove in a brief way those heretofore advanced.

Every theory advanced, except Sambon's has actually been demonstrated upon man or animals. This situation confronting us in the claims of scientific investigators is peculiar. That pellagra or symptoms of pellagra were produced cannot be doubted. I accept their results, but not their interpretations and conclusions. I agree that in the experiments on man the cause of pellagra was present. You will agree with me that there is one cause and one cause only. If I am able to show that the agents identified as the cause of pellagra were not present in every experiment in which pellagra was produced, then I claim that this evidence invalidates the maize theory. It is logical to presume that these experiments were performed in a city and that the subjects were not accessible to simulium reptans, which is essential to Sambon's theory. This, together with the development of pellagra in this country, explodes Sambon's theory. According to the statement of Dr. L. O. Howard, chief of the bureau of Entomology U. S. Department of Agriculture similium reptans does not occur in North America south of Greenland. I could also cite numerous cases that have developed in Atlanta and other towns of Alabama, Georgia and South Carolina. Fifty per cent. of our patients live in cities and some of them have not been near streams of water for years. The experiments attempting to demonstrate the maize theory are somewhat more difficult of disposition. It is necessary to know that these experiments were performed with spoiled corn, mould, organisms or with toxins obtained from them. These agents were either fed to the subject or injected. The demonstrations upon dogs were inconclusive and untrustworthy. In only one case was an erythema produced. In most instances the dog showed various symptoms of being sick. The greatest value that can be placed upon these demonstrations upon dogs is that the agent used made the dog present certain symptoms which were pathognomic of no certain disease. We conclude that in this condition the dogs (and men) were rendered susceptible more or less to attack by any disease.

A glance at the record of these experiments shows that the suspected agent used was not present in all of these demonstrations. From these demonstrations, because the disease was produced, we conclude that the cause of the disease was present but not recognized. I will try to show that in all probability there were three conditions present in every demonstration performed upon man, viz: 1st. A predisposing factor. 2nd. A secondary cause, and, 3rd. A primary or specific cause. The primary or specific cause (further evidence to be given later) is to be found in the fact that the subject ate or had previously eaten much adulterated olive oil or other fats to the same effect.

The secondary cause of the symptoms, which was used as an index to the production of the disease, erythema, etc., was the subjection of the skin of the subject to the action of the sun's ray.

A predisposing factor was the disturbance of physiologic balance by the introduction of deleterious or intoxicating material into the system. These conditions are of especial importance and are the only conditions present in each experiment.

The first condition I admit only as a contributory or predisposing cause. No need to discuss the effect of the sun's ray here. This phenomena is accepted by all observers.

In connection with the specific cause of pellagra, in the third condition I mentioned adulterated olive oil and other fats to the same effect. This statement pertains to semi-drying edible oils, or those used to adulterate olive oil, lard, and all more expensive edible oils and fats. These have been in use in France and Italy for centuries and cotton seed oil products which have come into extensive use within the last fifteen years. I propose to show that the eating of these oils and fats is probably the cause of pellagra.

These oils belong to a group classed as semi-drying oils. Chiefly and most extensively used are oil of maize, oil of sesame and oil of cotton seed. There are a number of oils of this class which may have been used for eating purposes or as adulterants. In order to show that my theory conforms to history and for convenience, I will discuss only oil of sesame and oil of cotton seed. Keep in mind, however, that all semi-drying oils suitable for eating purposes may have figured in the history of pellagra. The history of edible oils shows that oil of sesame (Benne oil, gingelly oil, til or teel oil) was known to ancient Persians and Egyptians and is esteemed by modern Arabs and other people of the East both as a food and as an external application to soften the skin.

For a long time the Chinese have ground cotton seed for cattle feed, and incidentally obtained a little crude oil for lubricating and illuminating purposes.

In 1833 a cotton seed oil mill was operated on a small island off the coast of Georgia. Previous to 1867 there were but seven mills in the country. The manufacture of cotton seed oil began on a large scale about 1870.

Much in history would be of interest which I think is unnecessary to relate here.

A casual glance will convince those interested that the cotton seed oil industry parallels the development of pellagra in the United States. Occasional cases may have occurred before this period from eating lard obtained from hogs fattened on cotton seed or through eating adulterated olive oil. (It is here necessary to mention that hogs may be fattened on cotton seed if the seed are properly prepared. Hogs so fattened in the fall of the year must be killed in the following winter or they will die the next summer. Lard rendered from these hogs is of the same chemical composition as cotton seed oil). From an historical standpoint this accounts for pellagra in every region for all time. I will now account for the geographical (?) distribution of pellagra in the United States. Those who are familiar with this disease know that they have seen many cases identical with pellagra which have no skin eruption. I believe that a visit to the hospitals will show there many cases who would develop the erythema upon exposure to the sun. Further, I believe many cases would not develop the erythema because of certain individual characteristics such as exert an influence upon and determine the other clinical features of the disease, in different individuals. Some idea of these individual characteristics may be had from the know fact that different people take on or lose different amounts of fat from the same diet. For some such individual characteristic everyone who is exposed to the cause of pellagra may not develop the disease. My investigations tend to show that the length of time between the beginning of eating cotton seed oil varies according to the preparation used and perhaps individual characteristics.

This period of time may be a few months or several years. Certain communities use almost entirely one brand. Others do not use cotton seed oil at all. In some sections the people began the use of Cotton Seed Oil when the price of lard advanced in 1908. Many people began its use for the sake of economy. Others because it is supposed to be more wholesome. Many people still buy and use lard. Pellagra in Georgia is limited to no section or class. Only individual characteristics determine immunity. In Italy and France if there is localization I believe it can be explained by the general health of the section, by the habits and dietic customs and by the financial status of the various sections (ability to buy food). Its mild and more chronic nature in France and Italy differs from the more acute and severe course in Georgia in degree only. The difference in cotton seed oil and the semi-drying oils of the East coincides with the course of the disease.

I will now define the disease and show how the cause probably acts in producing the chief clinical features.

Pellagra is a disease due to eating semi-drying oils in quantities that cannot be normally disposed of, in which case they are deposited as fat foreign to the human organism and in their final disposition by oxidation there is developed a series of end products that act in a deleterious manner upon all the cells and intercellular tissues of the body.

(There are no characteristic symptoms common to every case of pellagra—this is readily understood when the nature of the disease is explained). In order to make this clear it will be necessary to go into the chemistry of the semi-drying oils and the human fats.

The chief ones of this group are oil of maize, oil of sesame, and oil of cotton seed. In addition to the normal glycerides of the fatty acids found in the human body this group of oils contain glyceride of linolic acid. Glyceride of linolic acid (linolin) is the constituent of the group that places them in the semi-drying class. It is the drying constituent of these oils. Its drying property is due to the facility with which it oxidizes. When it is brought into contact with an oxidizing agent like permanganate of potash and haemoglobin it takes on oxygen and ultimately forms a series of volatile acids and aldehyde—of these oils, that of cotton seed contains the greatest amount of linolin. sesame oil a smaller amount and maize oil the least amount.

When linolin is oxidized linolic acid is formed. Linolic acid readily combines with the alkalies, forming salts which undergo oxidation even more readily than linolin or linolic acid. Also linolin has a strong affinity for sulphur and forms a compound which does not oxidize.

The oxidation products are the cause of pellagra. By preventing the formation of these products the occurrence of the disease is prevented or the progress stops.

It is, perhaps, true that food containing linolin may be taken, within certain limits, and produce no deleterious action. This limit is probably restricted only by the sulphur present with the foods and in the body. When this sulphur is exhausted the pyysiological limit is reached. Now when this physiological limit is reached the linolin is deposited in all the regions common to fatty deposits.

The more or less rapid oxidation may be an explanation of the various contradictory symptoms.

A more complete knowledge of these end products will probably throw a clear light on the pathological anatomy. Their nature is well enough known, however, to warrant the statement that they can produce the symptoms.

I wish here to submit a reaction which for lack of time I have not demonstrated, but am informed by analytical chemists and led to believe by a statement from Analysis of Oils., etc., by A. C. Wright, can and does take place. Wright says that linolic acid is oxidized by alkaline permanganate to sativic acid, azelaic or linusic acd and ultimately isolinusic acid, and aldehyde according to the strength of the solution. This reaction takes place in the presence of the oxygen of the air, but more slowly. As the conditions in the human body are the same, blood containing oxyhaemoglobin, I can see no reason why the same reaction does not occur. We have in this deposited fat (linolin) a condition where the law of mass action may also apply.

Read on page 1374 (continued description of aldehyde from page 1373) U. S. Dispensary, 19th edition, a description of aldehyde: "It is rapidly converted into acetic acid upon exposure to air. It boils at 71.6 degrees F. It possesses marked putrescent properties. It appears to paralyze the vagi though its cardiac action is feeble. Upon the resiration it exerts the most powerful influence, in small doses quickening it, in large doses depressing it. The temperature is much diminished—applied locally aldehyde is very irritating.

I believe that the clinical features and post mortem findings give evidence that this does occur. Observation will show the effect of aldehyde upon the respiration, heart and skin. Prof. Osler has stated that the skin symptoms are accompanied by suppuration. This is not true unless it is favored by treatment. With the rapid destruction of fat the characteristic atrophied appearance of the skin and tissues at the site of the erythema appears. This returns to normal when the erythema disappears and the patient takes on weight. We see statements that pellagrins improve upon the appearance of the eruption. They may do so when the rapid oxidation of fat (linolin) ceases. When they consult a physician who puts them on a diet or they go to a hospital where semi-drying oils are excluded from their diet. When they are restricted to bed and from the sun. There is no prodrome in pellagra, the sun's heat and ray alone promote rapid oxidation of the fat (linolin), which ceases only when the fat is all oxidized. When the patient is lean of hand the erythema is slight because of lack of fat to oxidize. If the hands are exposed during a warm period in Dec. the erythema appears. It may appear at any time of the year provided the sun is hot enough and there is a layer of fat. It takes 4 or 5 months for the fat to be deposited after it has been destroyed (on the hands in the spring or fall). This completely explains the spring and fall exacerbations. During the winter there is improved digestion and the patient is more vigorous; the time from the fall exacerbation to spring is longer, therefore more fat is deposited than during the summer, hence the spring exacerbation is more severe than the fall. Aldehyde or some of the volatile acids in their local effect account for the burning, pain, loss of sensibility.

According to this theory we would expect to find symptoms in these regions where the fat is deposited in thin layers and a rich blood supply. This is the case in every instance. example, mucous membranes when there is a rich active blood supply are affected; serous membranes are not.

The condition developed in the blood vessels in the skin and probably due to irritation, together with the fact that the action of the sweat glands are inhibited explains the slight temperature which is often present even in mild cases. I could go on, but believing I have furnished enough evidence to awaken investigators who will throw more convincing light on this, the greatest problem relating to the health of the whole world.. So far, I have made no allusion to functional changes in Pellagra. In a paper read before the American Gastro-Enterological Association in Philadelphia April 19th, Dr. Johnson gave tabulation and comIplete report of physiological perversion in these cases. I refer the reader to this paper, published in the Virginia Medical Semi-Monthly and Mobile Medical Journal for further evidence and application of these facts.

I will present evidence in the way of demonstrations on cattle and hogs which is common knowledge.

Suggestive information is to be had by the example of the cotton seed fed hog given above. It is a well known fact among farmers of this section that raw, fresh cotton seed fed to a hog will kill him in a few days, but if boiled or half-rotten seed are fed, the hog will take on fat. The hog fed on raw seed probably dies on account of intestinal obstruction produced by the impaction of accumulated and swelling seed. When these seed are boiled or half-rotted the hull softens by taking on water which admits more thorough mastication and digestion of the seed and contents. Also the swelling takes place before entering the stomach. The hog fed on the boiled or rotten seed is usually killed in the winter; if not, he will die next summer. It would be of interest to know if this hog would live longer if the cold weather continued or he was kept out of the sun. In years past cows were fed boiled cotton seed, but this feeding was during lactation, which may account for the immunity if there was any immunity. As far as I can learn cows of all cattle and animals are immune and then only during lactation.

To sum up the evidence we have a vast amount of proof that the semi-drying oils cause pellagra, nothing contrary. The strongest of this is that after discontinuing the use of these oils the disease does not recur. The relief given when sulphur is administered. The development and progress of pellagra in the United States has kept pace with the use of cotton seed oil. The universal use of these oils as a food by pellagrins. The symptoms can all be explained by the acceptance of this theory. The result if fed to animals. Linolin, which is foreign to the human organism can be demonstrated in the human body after the ingestion of cotton seed oil.

In the treatment heretofore given for pellagra, we have found little that will aid us in combatting the distressing symptoms present in the majority of cases. The treatment here proposed has been worked out after failure to give relief by using classical remedies. After this failure, we began a consideration of a departure. Our defeat in these cases taught us a lesson. From this experience, arose ideas of etiology, pathology and treatment.

It is acknowledged that the treatment heretofore suggested is empirical. The results of this treatment are uncertain and indefinite. Confidence in our remedies in the treatment of a condition is a material aid in getting resuts. In the majority of cases of pellagra, even though not insane, there is depression, despondency and melancholia. You have a mental state to impress. This impression is best made by a candid statement based upon honest conviction. Where we have accurate remedies and methods, we can so reassure these sufferers as to lift a shadow of despair which is so characteristic. How much or how little this mental impression has to do with subsequent results cannot be estimated. Certainly, it is more conducive to good results to have our patients and his associates relieved of an atmosphere of doom. As this is to be a practical statement of a practical treatment, we will proceed from this view point:

First: We impress our patients that pellagra can be cured. We assure them this will take time.

Second: We inform them that when the pellagra is cured, we will have to deal with the damage it has done together with, in some cases, a chronic predisposing factor.

In relieving the mental strain (not insanity, we will deal with this symptom later), we put our patents in a fighting attitude, prepare him for a hard fight that is necessary for results, and urge his co-operation to the end. In our observation, these patients have been in poor health for years and will cherish the idea that they can, by persistent effort, be enabled at least to live in comparative comfort.

As to medication, I wish to state that the remedies are given with a definite idea as to the nature of the disease.

We all agree that pellagra is a sort of intoxication. With a knowledge of the source of any intoxication, we have two lines of attack open to us; by a specific agent we eradicate the cause and protection of the organism against deleterious effects of the poison. I have already shown that sulphur antagonizes the oxidation of the Linolin. Calcium given in combination with it is especially indicated, hence: as a specific agent, we give calcium sulphide. Given in doses of one-half, to two or more grains three or more times daily it shows its effect in a few hours. In fact, the results are so prompt that I would feel like claiming specific action without a knowledge of its chemical action. We began the use of calcium sulphid eighteen months ago and have never been disappointed in the results. Under its influence I have seen the stools reduced to normal: the redness of the tongue fade; the eruption on the hands disappear. The intense burning and pain so prominent in some cases yield readily (usually in less than twenty-four hours). One case in whom the eruption had been continuous for twelve months, although being treated by arsenic was relieved of suffering in twelve hours, was out of bed in three days, and had no erythema in ten days. In all cases, this drug is all that is necessary to relieve aggravated cases of simple pellagra. That it does not relieve all cases is due to the fact that some are complicated by a long standing gastro-intestinal lesion which antedates the onset of pellagra by a more or less period of time. In these cases, we have a totally distinct condition to treat. Some of these cases were under treatment several years previous to development of pellagra, suffering from various affections of gastrointestinal origin, which was probably a predisposing factor in the development of pellagra.

So, believing that in calcium sulphid we have an agent capable of combatting the deleterious action, and enabling the organism to rid itself of linolin, we have only to deal with these complicated cases which need special symptomatic treatment in addition.

I believe our failure in America to give relief has come from a lack of experience in dealing with a new condition. We have failed to take into consideration the important predisposing and complicating factors. Pardon me if I try to escape the criticism of some who leave much to be taken for granted.

I insist that success in treatment means knowledge of indications, the proper remedy, accurate dosage and methods of administration. (Some to whom I have suggested this treatment gave one-sixth grain calcium suphide and reported failure. In writing prescriptionswe never specify a certain manufacturer's article when it can be avoded, but from experience with this drug, we have been forced to be very careful with this product. Before you prescribe it, you should find out whether or not it is soluble as many of them are not. The best way to administer it is in 1-8 or 1-6 grain pills. They may be given in a capsule as many as you like.

. If there is belching, it will be disagreeable. This can be avoided, by giving an enteric pill. In fact, cases in which there is normal or hyperacidity, we believe the enteric pill should always be used.

In order to make this clear, we will mention each symptom and complication.

Some patients are given electricity, galvanic, faradic, galvanofaradic or high frequency according to well known physiological indications. In exhausted cases, strychnine or sparteine a needed.

In the paper referred to above, Dr. Johnson gave several prescriptions after further observations, we advise against the use of any oxidizing agents during the presence of linolin within the body. In view of the further lights thrown upon this subject, readily see that all drugs such as urotropin should be avoided except as indicated after the linolin has been removed.

We advise against ointments, as they aggravate the erythema. In fact, we believe the most erythema which is so distressing, is due to some local application applied by the patient before he consulted by a physician. In our observation, the eruption is always dry and non-suppurating unless so treated.

Insomnia may need relief during the first few days of treatment. However, it is relieved when the bowels are open and the kidneys act well.

The same is true of insanity—in all cases during the first four years. (We have seen no cases of longer duration.)

Constipation is best relieved with castor oil, which also seems to benefit the nervousness, depression and insanity. This drug should be given in mornings daily, in one to four drachm doses or sufficient amount to procure one to three good stools daily.

Pains may occur in the stomach or bowels. When in the stomach, give Paregoric 1 dram., Sod. Biccrb. 1 dram in one-half glass warm water and apply hot water bottle. If this does not relieve, use gastric lavage or hypodermic of morphia.

For pain in the bowels, give enema of saline solution. When pain is not relieved by enema in diarrhoea, give bismuth and paregoric 1 dram (aa). Myasthenia cardia present in some cases and always a source of danger calls for special mention and should be carefully guarded in cases suffering from nausea and vomiting or diarrhoea. In such cases, thirst is great on account of drainage and loss of fluid from vomiting. Rest in bed and restriction of fluids should be instituted. Solid food, steak or chicken with crackers or toast (dry) should be given every two hours. Water should be given in doses of four ounces with the food and at no other time until the nausea and vomiting cease. Gradually increase the amount of food and fluids.

Diarrhoea which is present in a large per cent. of cases, is easily relieved when it comes on after the onset of pellagra. Those cases suffering with diarrhoea before the onset are sometimes obstinate and must be considered apart from this disease. Others are suffering from an independent disease and must be treated accordingly.

In these cases we make a complete examination of the gastric secretion and fecal matter. With a duodenal pump we obtain a specimen of bile and pancreatic secretion; this usually gives a clue to the cause. Should the diarrhoea continue after three or four days observation, we follow a line of treatment indicated by the examination.

Further discussion of this symptom would carry us into every phase of Gastro-enterology and is not pertinent to this paper; hence, we have only a few suggestions to offer.

Zinc sulpho-carbolat is used in putrefication and fermentation. In all cases, having diminished gastric secretion hydrocloric acid and css. pepsin is invaluable. Bismuth and tannigen meet an indication in profuse serous diarrhoea which is alone present in some cases. In diminished secretion of bile and lack of biliary alkalies, calomel will be of benefit. Two cases complicated with syphilis were given a grain of calomel in a. m. and p. m. for diarrhoea.

Diet: we also consider a purely gastro-intestinal problem and will mention a few important facts to be followed while getting the medicinal effects.

In the beginning, we place most patients on a milk and egg diet. In one or two weeks, as the gastro-intestinal symptoms permit, we allow a liberal diet.

For obvious reasons, those suffering with nausea and vomiting and some with diarrhoea fare best on chicken, steak, crackers and toast.

In conclusion, I wish to advise against the use of fats and oils of every kind except castor oil. This warning is not without foundation, and in the light of my investigation, it is justified.

Olive oil, which in this day and time is synonymous with cotton seed oil is to be prohibited.

Patients treated as above, who have carried out the treatment and suggestions as to prophylaxis, have not relapsed. True some of these cases are not yet strong and sound. We do not expect to repair all the defects in dyspeptics, or those who have been suffering from some other pre-existing disease of five, ten, fifteen or twenty years standing while treating a vicious condition like pellagra. In many cases, after relieving all traces of pellagra, we have to deal with a condition far more chronic and resistent in addition to the damage done by months and years of intoxication.

(Note.—I wish to give credit to the various standard works which have been freely used and drawn upon in elaboration of this theory.)

55 E. Harris St.

